# ‘Why do we have to be the gatekeepers?’ Australian general practitioners’ knowledge, attitudes and prescribing intentions on e-cigarettes as a smoking cessation aid

**DOI:** 10.1186/s12875-024-02292-w

**Published:** 2024-02-07

**Authors:** Melis Selamoglu, Bircan Erbas, Hester Wilson, Chris Barton

**Affiliations:** 1 Department of General Practice, School of Public Health and Preventive Medicine, Monash University, Melbourne, Australia; 2 Department of Public Health, School of Psychology and Public Health, La Trobe University, Melbourne, Australia; 3 Population and Community Health, South Eastern Sydney Local Health District, Sydney, Australia; 4 School of Population Health, University of New South Wales, Sydney, Australia; 5 Centre for Primary Health Care and Equity, University of New South Wales, Sydney, Australia

**Keywords:** e-cigarette, Smoking cessation, General practitioner, General practice, Primary care, Public health

## Abstract

**Background:**

A significant policy change impacting the availability of nicotine for use in electronic cigarettes (e-cigarettes) in Australia took effect from October 1, 2021. This change meant that nicotine containing liquids for use with e-cigarettes would only be available by prescription from a medical practitioner as part of a smoking cessation plan. This study aimed to explore general practitioners (GPs) perceptions about the role of e-cigarettes, and understand factors informing their intentions to prescribe e-cigarettes as part of a smoking cessation plan.

**Methods:**

In-depth semi-structured interviews were conducted with thirteen GPs. Purposeful sampling was used to recruit participants. Interviews were audio recorded and transcribed verbatim. Thematic analysis was used to classify, describe and report themes in the data. QSR NVivo was used to aid coding, thematic analysis and retrieval of quotes.

**Results:**

Participants had diverse views on recommending and prescribing e-cigarettes as smoking cessation aids to patients. Some participants were willing to prescribe e-cigarettes to patients if other methods of smoking cessation had not worked but there were concerns, and uncertainty, about the safety and efficacy of e-cigarettes for smoking cessation. There was poor understanding of the current policy and legislation about e-cigarettes in Australia. Mostly the participants in this sample did not feel confident or comfortable to prescribe, or have discussions about e-cigarettes with patients.

**Conclusion:**

The participants of this study held diverse attitudes on recommending and prescribing e-cigarettes for smoking cessation. Clarity in guidelines and consumer product information are required to enable GPs to provide consistent and accurate advice to patients that wish to use e-cigarettes as a smoking cessation aid.

**Supplementary Information:**

The online version contains supplementary material available at 10.1186/s12875-024-02292-w.

## Introduction

Australia is known as one of the world’s leading nations in tobacco control. It has one of the lowest smoking rates worldwide, with just 11.2% of people aged 14 years and over reporting daily smoking in 2020-21 [1, 2]. This success has been attributed to the implementation of tobacco control policies such as smoke-free laws, anti-smoking mass media campaigns, graphic health warnings, regular increases in tobacco taxes, bans on advertisement and promotional materials, and the introduction of plain packaging [2, 3]. From September 2011, nicotine products other than transdermal or oromucosal use have been classified as prescription only (Schedule 4) medicines for human therapeutic use and as dangerous poisons (Schedule 7) for non-therapeutic use in the Poisons Standard of the Therapeutic Goods Administration (TGA) [4–6]. Recently, a policy was introduced in Australia to limit a rapid increase in the number of electronic cigarettes (e-cigarettes) users through regulation of nicotine containing e-cigarettes.

E-cigarettes or electronic nicotine delivery systems (ENDS) are battery powered products that heat e-liquids to produce an aerosol that can be inhaled by the consumer and may be harmful to the health of the user [7–9]. E-cigarette devices come in two formats, ‘open’ which are refillable and ‘closed’ which are disposable or refilled using cartridges [8]. E-cigarettes are increasingly being marketed to both young people (people who never smoke) and older people who smoke. They are promoted as a safer, cheaper and less risky option to smoking and have been promoted as smoking cessation treatments although no e-cigarettes on the market have been approved as therapeutic goods [10, 11]. Although these products do not contain tobacco, they do contain nicotine, propylene glycol, glycerine, flavouring agents and other chemicals that can be harmful to health when heated and inhaled [8, 9, 12].

A significant change to policy impacting the availability of nicotine for use with e-cigarettes in Australia took effect from October 1, 2021 [4]. This change meant that nicotine containing liquids for use with e-cigarettes will only be available by prescription from a medical practitioner. In June 2020, the Health Minister announced that e-cigarettes would only be available by a prescription from a medical practitioner for Australian individuals who smoke to be able to use e-cigarettes as a smoking cessation alternative [5]. This policy clarification was to address the rapid increase in the use of e-cigarettes by young adults and teenagers in the US and Australia [5]. This provides an opportunity for health care providers and general practitioners (GPs) in particular, to discuss use of e-cigarettes and provide information and support for smoking cessation with patients who use, or are interested in using, e-cigarettes. However, little is known about Australian GPs preparedness to have these discussions with their patients and their perceptions of e-cigarettes as an alternative to existing smoking cessation therapies.

Currently, there are three main pathways Australian GPs can use to prescribe nicotine containing e-cigarettes to patients as a third-line treatment in combination with behavioural support and/or pharmacotherapy medications to people who smoke and have been unable to quit successfully using pharmacotherapy treatments (e.g. nicotine replacement therapies (NRTs), varenicline and bupropion) [13]. These are the *Authorised Prescriber Scheme*, where GPs are able to prescribe nicotine containing e-cigarettes to an unlimited number of patients for up to 5 years and requires approval from the TGA for supply within Australia [4]. The *Special Access Scheme* allows for the importation and supply of nicotine containing e-cigarettes to only one patient on a case by case basis and requires TGA approval for domestic supply [4]. The *Personal Importation Scheme*, which allows for the patient to import up to three months’ supply of nicotine containing e-cigarettes per importation for personal use, but must not exceed 15 months’ supply in one year and does not require TGA approval [4].

The National Drug Strategy Household Survey (2019) conducted by the Australian Institute of Health and Welfare reported that amongst Australians aged 14 and over, 32% had tried e-cigarettes to help with quitting smoking [14] and 3.2% used them daily [1]. As for non-smokers, the most common reason for trying e-cigarettes was curiosity [14], 6.9% had tried e-cigarettes [1] and 1.4% were currently using them [14]. In addition, the general population of people who smoke that were surveyed perceive that e-cigarettes were cheaper and less harmful than standard cigarettes, tasted better, reduced the number of cigarettes smoked and stopped them from smoking regular cigarettes [14, 15].

In a recent systematic review, it was concluded that little was known about GPs preparedness to have discussions with patients about e-cigarettes for smoking cessation and that GPs intentions to prescribe e-cigarettes as smoking cessation treatments were unclear [16]. Outside of Australia in countries such as the US and the UK, GPs reported they lacked confidence in their knowledge of e-cigarettes to effectively communicate with their patients about their use, indicating that more evidence and information was needed, including their use as a smoking cessation therapy [17].

GPs have an important role in providing patients who smoke with information, support and recommendations, referral or a prescription for treatments to support them to quit smoking. Given the high degree of uncertainty about the place of e-cigarettes as a therapy to support smoking cessation in Australia, this study aimed to explore GPs perceptions about the role of e-cigarettes, and understand factors informing their intentions to prescribe or not prescribe e-cigarettes as part of a smoking cessation plan.

## Methods

### Study design

A qualitative descriptive study design [18] was used to explore and understand the perceptions of Australian GPs toward e-cigarettes as a smoking cessation treatment and to provide insights and context to survey data collected from a large sample of GPs about their knowledge, attitudes, beliefs and intentions to prescribe e-cigarettes for smoking cessation. Qualitative research has the ability to provide deeper insights and information to inform the development of policies and guidelines to support clinical practice and provide avenues for further research and investigation [18]. This paper has been prepared using the consolidated criteria for reporting qualitative research (COREQ) guidelines to guide the description of the study methods and reporting of findings [19].

The research is being undertaken as part of doctoral studies of the lead author (MS) who has a background in Health Science and a Masters in Health Policy. MS completed training within the Department of General Practice on qualitative interviewing and is supervised by academics with experience in qualitative research and respiratory epidemiology (CB and BE). The views of a general practitioner (HW) experienced in addiction and smoking cessation were sought and helped refine the interpretation of data. MS is a non-smoker and does not vape, nor has any affiliations with the tobacco industry or pharmaceutical companies. Throughout the study a reflexive research journal was kept as a record of the audit trail and to aid researcher reflexivity.

### Sampling and recruitment

Participants were drawn from a larger sample of Australian GPs (*n* = 246) who completed a survey study about their knowledge, attitudes and beliefs, social norms and intentions to prescribe e-cigarettes [20]. Initially the authors purposefully sampled participants from different states, gender and years of practice as these were important points of differences in the survey responses which we wanted to explore and understand in further depth.

Participants (*n* = 33) who provided a contact email and indicated a willingness to participate in the qualitative interview study were invited to participate in May 2022. From the 33 participants who provided their email addresses, eighteen did not respond, one accepted but did not attend the meeting and one declined. Interviews with *n* = 13 (39%) GPs were conducted throughout May and June 2022. From the thirteen GPs that were interviewed, three had registered to be an Authorised Prescriber and had prescribed e-cigarettes to patients. All interviewees provided verbal informed consent before participating in the study. Participation was voluntary and participants completing the interview were offered a $100 gift voucher as an honorarium.

Ethics committee approval for the study was provided by Monash University Human Research Ethics Committee.

### Data collection

One on one in-depth interviews were conducted by the lead author (MS) and were guided by a semi-structured interview guide which can be found in the supplementary file. The interview guide was developed following the approach of Minichiello et al. [21], with broad thematic areas for discussion identified from a review of the literature, guided by the Theory of Planned Behaviour (TPB) [22], and issues emerging from a preliminary analysis of survey findings [20].

The TPB encompasses three domains, the individuals’ *attitude* and behavioural beliefs towards the behaviour, *subjective norms* and the influence of social pressure and, *perceived behavioural control* which refers to an individuals’ ability to perform a behaviour based on control beliefs (i.e. skills, resources, time, money, opportunities) [22, 23].

All interviews were conducted online via Zoom^™^ and ranged in length between 25 and 60 min. The first three interviews were used to pilot test the interview schedule and were transcribed verbatim. Two authors, MS and CB, reviewed these transcripts and discussed the quality of the interviews, the scope of the content and need for any amendments. No major changes were necessary and the pilot interviews were retained for analysis.

The interviews included discussion of the role of e-cigarettes in clinical practice, beliefs, knowledge and attitudes about the use of e-cigarettes in regards to smoking cessation, the prescription of e-cigarettes to quit smoking and the confidence and comfort levels of GPs in giving advice to patients about e-cigarettes in smoking cessation.

### Data analysis

Interviews were audio recorded and the file was transcribed verbatim by a professional transcriptionist. Two authors (MS and CB) met regularly during the data collection phase to discuss the interviews and emerging themes. The analytic approach of Braun and Clarke [24] was used to analyse the data. MS initially read and re-read transcripts and immersed herself in the data. A preliminary coding framework was developed using QSR NVivo. This was discussed with the other authors and codes were organised into themes based on mutual agreement. Analysis of the data included both deductive coding guided by the TPB [22] and inductive coding. By the thirteenth interview no new concepts were emerging and authors agreed that further interviews would not be required.

## Results

### Study population

Demographic characteristics of participants are reported in Table 1. It can be seen the sample comprised GPs located in 5 states, the majority in metropolitan regions (*n* = 12). A balanced number of females and males were interviewed. Participants had been practicing on average for 19 years. Three participants worked in academia, two in addiction services and one in an Aboriginal health service.

**Table 1:** Demographic characteristics

		Total *N* = 13
Gender	Female	*n* = 7 (54%)
	Male	*n* = 6 (46%)
Years of practice	1–10	*n* = 4 (31%)
(min 4- max 46 years)	11–20	*n* = 4 (31%)
	21–30	*n* = 3 (23%)
	30+	*n* = 2 (15%)
State	Victoria	*n* = 4 (31%)
	Queensland	*n* = 4 (31%)
	New South Wales	*n* = 3 (23%)
	South Australia	*n* = 1 (7.5%)
	Western Australia	*n* = 1 (7.5%)
Region	Metropolitan	*n* = 12 (92.5%)
	Rural	*n* = 1 (7.5%)
Registered Authorised Prescriber	Yes No	*n* = 3 (23%) *n* = 10 (77%)

Participants reported they would use clinic software (*n* = 12) to record patients smoking status and all reported they asked their patients on their initial consults whether or not they were current or former persons who smoke.

Five main themes were identified: GPs attitudes toward e-cigarettes, concerns about safety of e-cigarettes, concerns of dual use and the gateway effect to other tobacco products, GPs understanding of policy and finally, mixed levels of confidence and comfort prescribing and discussing e-cigarettes with patients.

### Theme 1: GPs attitudes on e-cigarettes

There were diverse views among the participants interviewed toward recommending and prescribing e-cigarettes to patients. These mixed opinions arose from gaps in the participants’ knowledge about e-cigarettes as well as concerns they held about the availability and veracity of data on the safety and efficacy of e-cigarettes. Behind these diverse attitudes were concerns about the quality of evidence underpinning recommendations about the role of e-cigarettes as a smoking cessation therapy.

Some participants described how they were adamant they would not recommend e-cigarettes, such as participant #2 and #8 who stated:

*“I won’t recommend them, I have not recommended them.”* (GP#2, M, 6 years of practice).

*“[There is] no situation in which I would recommend an e-cigarette. I am not going to recommend it to anyone.”* (GP#8, M, 40 years of practice).

However, others were willing to consider them if they had further evidence that they help patients quit smoking, with particular interest in groups of patients such as “older established smokers” (GP#13, F, 25 years of practice) that haven’t been successful quitting using other available treatments.

*“As a last resort to my older patients who are quite desperate to quit and have failed using all other methods.”* (GP#4, F, 7 years of practice).

*“I don’t know if I would [recommend e-cigarettes] until more information and evidence comes out.”* (GP#11, F, 17 years of practice).

Some participants who had tried established treatments to help their patients cease smoking without success, were willing to recommend e-cigarettes as a “third line option” (GP#12, M, 19 years of practice) and as a “last resort” (GP#1, M, 46 years of practice) to patients that have “tried varenicline, bupropion, NRTs, counselling, behavioural support and hasn’t worked” (GP#7, M, 23 years of practice).

The attitudes held by participants reflected their knowledge and confidence in their understanding of e-cigarettes. Participants stated they had “limited” (GP#4, F, 7 years of practice) to “adequate” (GP#3, F, 25 years of practice) knowledge about e-cigarettes, whilst others believed they had enough, “basic knowledge” (GP#2, M, 6 years of practice; GP#9, F, 4 years of practice; GP#10, F, 15 years of practice) to offer advice if patients asked. Some participants mentioned they haven’t had patients ask them about e-cigarettes to quit smoking and hence, didn’t know much about the topic.

*“Probably not really [having enough knowledge]. In practice a lot of things are stimulated by having a patient ask us then if we’re good doctors we’d go and do some homework, so we can advise them. I’ve got a bit of understanding but I haven’t really, because I haven’t had anybody ask me about it.”* (GP#1, M, 46 years of practice).

A few participants mentioned they remained uncertain about how to prescribe and decrease the nicotine dosage after patients had started using e-cigarettes. They desired more information on how to do so.

*“I just don’t think as a doctor we are really knowledgeable or trained in how to talk to people about milligrams of nicotine that is equivalent in their vaping liquid and how much they should be reducing that by over what period of time. There is just no information on that.”* (GP#6, M, 11 years of practice).

*“When it comes down to the individual doses, duration of being on a certain dose, before you step up or down. That part of it I don’t think I have as much knowledge.”* (GP#9, F, 4 years of practice).

In order to enhance GPs level of knowledge, participants wanted further materials and resources available to them. These included updated, easy to read guidelines on e-cigarettes from the Royal Australian College of General Practitioners (RACGP) and TGA. Some participants also mentioned they would like to receive training as long as it was not a long and time intensive training session. They would prefer the session be pre-recorded for them and be available online to view in their own time and when they felt the need to watch it as a reminder. A simple one-page summary or an infographic of the pro’s and con’s on e-cigarettes was also preferred as well as, a quick snapshot on the types of devices, flavours and strengths of nicotine e-liquid to help GPs prescribe e-cigarettes to their patients.

Some participants mentioned they would like to see the “latest up to date evidence” (GP#4, F, 7 years of practice) on e-cigarettes as smoking cessation aids to help guide them in their current practice.

*“I’d like to see more evidence that compares it to other smoking cessation aids, but I haven’t seen any or heard of any good evidence yet.”* (GP#3, F, 25 years of practice).

*“Evidence for cessation of smoking [using e-cigarettes] over a three-month program with the following doses and frequency of use, something that looks a little bit scientific.”* (GP#8, M, 40 years of practice).

Participants gained information and knowledge on e-cigarettes through medical journals, social media platforms and guidelines from the RACGP. This information helped shape their attitudes toward the role of e-cigarettes as part of a smoking cessation strategy.

### Theme 2: Safety sits above all in considering a role for e-cigarettes in smoking cessation

Many participants expressed optimism about the role that e-cigarettes could potentially have as smoking cessation treatments. Some participants described their belief that e-cigarettes were “much safer” (GP#6, M, 11 years of practice; GP#8, M, 40 years of practice) and “less harmful than regular cigarettes” (GP#9, F, 4 years of practice; GP#13, F, 25 years of practice).

*“I think they are a good tool [e-cigarettes] providing we accept that there are unknowns to it, but I think it is a good tool [e-cigarettes] to get people to stop smoking. If it’s used appropriately as smoking cessation it is a powerful tool [e-cigarettes].”* (GP#6, M, 11 years of practice).

*“I think they [e-cigarettes] have a role that’s sort of second or third line after the other options which we kind of know the safety data on. My understanding is that they’re [e-cigarettes] more effective, or have been shown to be more effective than NRTs.”* (GP#9, F, 4 years of practice).

*“The role that I see them [e-cigarettes] as, it’s automatically taking out a lot of really harmful carcinogens and it’s basically another form of NRTs that has the cultural, psychological, and behavioural link that gum, lozenges and patches don’t have. I think it’s a great option for smoking cessation.”* (GP#5, F, 9 years of practice).

However, others expressed concerns about potential harmful effects e-cigarettes could have on the body and not knowing the long-term health effects they may produce in the future.

*“Concerns about harms and not knowing if they [e-cigarettes] actually do help people stop smoking enough to be worth recommending them to use.”* (GP#1, M, 46 years of practice).

*“There’s potential harm still if there’s additives to the vape, like flavours and fragrances. There could still be other chemicals there that we just don’t know enough about.”* (GP#3, F, 25 years of practice).

### Theme 3: Concerns of dual use and gateway effect to other tobacco products

There was apprehension about the use of e-cigarettes for smoking cessation, “as it has the potential to become a window or a door to other products and a pathway to smoking” (GP#2, M, 6 years of practice). Making the devices more accessible and more visible was considered by some as providing a gateway to groups at risk of taking up smoking like young people.

*“I think that’s a huge risk [dual use], and I think it’s already happening. You can see socially that people are more likely to interchange their use, depending on what’s available at the time.”* (GP#4, F, 7 years of practice).

*“It’s a gateway to an unnecessary addictive behaviour that isn’t going to do anyone any favours. Even if they [young people] just vape and do nothing else, that’s worse for them if they didn’t vape, and that’s enough harm for me to feel very uncomfortable about what’s happening in young people who are being recruited to vaping.” *(GP#13, F, 25 years of practice).

Participants expressed concerns on the various types of flavours available to young adolescents and the potential groups the tobacco industry is trying to target.

*“It’s [e-cigarettes] easily accessible, young people would probably be thinking it’s kind of cool – different colours, different flavours.”* (GP#4, F, 7 years of practice).

*“The biggest thing is that I think it is being used to appeal to the adolescents and younger markets who aren’t smokers. It’s like cigarettes all over again. It’s an image thing. It’s because of the candy flavours.”* (GP#6, M, 11 years of practice).

### Theme 4: ‘A bit of a guess’ – GPs understanding of policy was limited

Understanding of current policy about e-cigarettes in Australia was limited amongst participants. Some stated they knew “very little” (GP#6, M, 11 years of practice) or their understanding of it was “not very good” (GP#1, M, 46 years of practice) or they didn’t know the “specific ins and outs” (GP#4, F, 7 years of practice), or had very little idea of the current legislation.

*“My understanding of the current policy is that it’s similar to smoking, there’s no smoking including vaping in cars with kids and then in public places and inside. In terms of e-cigarettes it’s illegal to supply it to a minor, illegal to have nicotine liquid without a prescription.”* (GP#10, F, 15 years of practice).

A couple of participants had formed a *‘personal’* policy position, for example.

*“I think the policy for me is, it’s not yet established in terms of what we are going to do with this phenomenon. It is misinforming, it is changing, there is differences in the opinions between the policy makers across the country, between states and territories. It has created some confusion for service providers including clinicians.”* (GP#2, M, 6 years of practice).

*“My policy is that there’s no evidence to recommend it’s use in smoking cessation.”* (GP#11, F, 17 years of practice).

Only a few participants had prior knowledge of the current legislation.

*“I understand it’s meant to be only on a doctor’s prescription that can be compounded or imported. But there’s not any TGA listed specific liquids or devices that are really available.”* (GP#7, M, 23 years of practice).

*“My understanding is that the products are not TGA approved, but there was a desire to regulate the industry, and so, prescriptions are required to legally purchase them or legally import them.”* (GP#13, F, 25 years of practice).

A small number of participants expressed their concerns and frustrations on being gatekeepers to e-cigarettes. They “wished they didn’t have to do this” (GP#12, M, 19 years of practice) and tended to disagree with it. They didn’t see “why this needs to be a GPs problem and that doctors don’t have a role here” (GP#6, M, 11 years of practice). One participant had seen “a lot of people [other GPs] voice discomfort at being put into a gatekeeper role” (GP#7, M, 23 years of practice).

Further discussions took place about legal risks.

*“I think the ownership of me prescribing it [e-cigarettes] and taking on that risk, is a massive negative. That’s a big barrier because why would anyone want to risk legally their career and their potential for legal ramifications 15 years from now because it’s an unknown quantity that has been mandated but, it can only come from me which is really frustrating?”* (GP#5, F, 9 years of practice).

*“I know that a lot of other GPs were really wary about prescribing or endorsing e-cigarettes and didn’t feel like they were I suppose medico-legally protected if they did that. A lot of GPs don’t feel very confident about them.”* (GP#9, F, 4 years of practice).

One participant suggested having consent forms available for patients to sign and read before giving a prescription of e-cigarettes to cover any future legal ramifications.

### Theme 5: Mixed levels of confidence and comfort prescribing and discussing e-cigarettes with patients

Participants stated they felt “fairly confident” (GP#7, M, 23 years of practice; GP#9, F, 4 years of practice; GP#10, F, 15 years of practice; GP#12, M, 19 years of practice) or “moderately confident” (GP#3, F, 25 years of practice; GP#6, M, 11 years of practice) but others were “not confident” to answer patient questions about e-cigarettes (GP#1, M, 46 years of practice; GP#2, M, 6 years of practice; GP#11, F, 17 years of practice).

The sample include participants that ranged in their comfort and confidence about prescribing e-cigarettes. Some GPs didn’t feel comfortable (GP#7, M, 23 years of practice; GP#11, F, 17 years of practice) to do so as they “hadn’t had suitable patients” (GP#3, F, 25 years of practice; GP#6, M, 11 years of practice). Others didn’t feel comfortable due to lack of knowledge and the need for further information.

In order for participants to feel comfortable to recommend e-cigarettes for smoking cessation to patients they suggested having further training and information available to them when they needed it, particularly, a one-page summary of pertinent information.

Others wanted to see more information from the TGA on the types of e-cigarette devices in addition to information on recommended doses for nicotine e-liquids that GPs should prescribe as they lacked knowledge in this area.

*“From the TGA, it would be nice to see more about recommended doses, maybe even a guide to which pharmacies are stocking them and are registered if that’s a requirement.”* (GP#9, F, 4 years of practice).

*“Sometimes patients will try one vape but they don’t like that device. Some more specific guidance around well, if this device doesn’t work for you, why is that, maybe you need a higher concentration, or maybe this device doesn’t suit you, what device next?”* (GP#10, F, 15 years of practice).

## Discussion

This group of Australian GPs held mixed views about recommending e-cigarettes as smoking cessation aids to patients, and the prescription of e-cigarettes for smoking cessation. These mixed views were driven by a perceived lack of authoritative information about e-cigarettes and nicotine liquids from trusted authorities, uncertainty in the data on the safety and efficacy of e-cigarettes, and limited understanding about policy regulating e-cigarettes in Australia. Some participants were willing to recommend e-cigarettes to patients, but primarily in the context of a second- or third-line treatment when other options had not worked. They required more information on how to prescribe and then step down the nicotine dosage after patients had started using e-cigarettes. Some participants believed e-cigarettes to be a safer alternative to regular cigarettes, whilst others disagreed. Participants expressed concerns that e-cigarettes may contribute to broad acceptance and use of e-cigarettes outside the clinical indication for smoking cessation and that they could create a gateway effect to cigarette smoking or dual use. Participants that knew more and were more confident in their understanding felt better prepared for patient discussions about e-cigarettes and were comfortable to prescribe e-cigarettes.

The intentions of GPs to prescribe a medication can be explained by theories such as the TPB [22]. The TPB has been used extensively to predict behaviour in various areas such as smoking cessation [25, 26], drugs [27], alcohol [23] and other behavioural contexts including prescribing behaviour [28, 29]. Participants in this study showed that those who believed e-cigarettes to be safer and less harmful than regular cigarettes, had better knowledge about e-cigarettes, felt comfortable and confident to have discussions with patients about e-cigarettes and had a positive attitude towards recommending e-cigarettes. These participants had greater intention to prescribe e-cigarettes as smoking cessation aids.

The current guidelines from the RACGP state that e-cigarettes may be an alternative option to recommend to patients, with behavioural support, only if people who smoke have tried to cease smoking using first line therapy methods and have failed to do so [13]. Mostly, participants in the current study held views in line with this recommendation from the RACGP [13], but others held quite staunch perspectives against their use. This could be because some participants require further scientific evidence showing the effectiveness of e-cigarettes for smoking cessation compared to other alternatives as well as, the lack of knowledge and confidence to prescribe e-cigarettes. Participants are willing to prescribe e-cigarettes only if further research and authoritative evidence showed that e-cigarettes can have a role in smoking cessation. Others believe the use of e-cigarettes merely is switching from one tobacco product to another, continuing nicotine dependence.

In some international studies, family physicians show to have lack of knowledge and training as well as, diverse opinions about the safety and effectiveness of medical marijuana [30–32]. Future suggestions recommend that family physicians should be provided with training programs to increase their knowledge and address their concerns around the safety of medicinal cannabis [30–32]. This is in line with our findings in regards to e-cigarettes as smoking cessation aids, and has been suggested by our participants that educational materials and resources are beneficial to increase knowledge around e-cigarettes as a form of smoking cessation.

Our sample of Australian GPs had various attitudes and views on recommending e-cigarettes as smoking cessation aids which is similar to findings from family physicians in the US [33, 34]. Like US family physicians, some Australian GPs in our study would gain confidence in their recommendation to use (or not use) e-cigarettes to patients if scientific evidence, with greater certainty in its conclusions, demonstrated e-cigarettes to be effective in smoking cessation. More recent, well conducted systematic reviews [35] completed since we undertook these interviews provide evidence of the efficacy of e-cigarettes in smoking cessation and so, it will be interesting to see if this evidence has changed the perspectives of these GPs. Other studies have also identified that GPs report they would be happy to endorse e-cigarettes to patients if research such as clinical trials and systematic reviews showed e-cigarettes to be as effective as other smoking cessation treatments [34].

There were many participants in our group who indicated they did not yet have sufficient knowledge to answer patient queries about e-cigarettes. In Australia, there is an ongoing debate about their place within the broader set of medicines and therapies to support smoking cessation, as has been found internationally [36]. Clarity in guidance and accessible, GP friendly resources are needed from trusted organisations such as the TGA and RACGP to support GPs to have these discussions with their patients if asked.

Our findings are consistent with international literature [16, 33, 34, 36–39] that more empirical evidence is needed so GPs can provide accurate and effective advice to patients about the use of e-cigarettes as a smoking cessation treatment. A one-page summary or an infographic of the pro’s and con’s on e-cigarettes or, a quick snapshot of pertinent information that could be handed out to patients for information was desired. Some GPs in similar studies saw e-cigarettes to be less harmful and safer than regular cigarettes [33, 34, 36, 37, 39], consistent with our findings, but others disagreed with this. In the US, there are concerns among family physicians about safety and thus, reluctance to recommend them as smoking cessation aids [33, 34]. Discussions around concerns of e-cigarettes potentially being a gateway to conventional cigarettes and other tobacco products was an issue among our sample of participants, and of GPs in prior studies [34, 38, 39]. This impacts the perception of risk that can influence intentions for clinicians to prescribe a treatment. Many believed young adults were being targeted by the tobacco industry, and that use of e-cigarettes should not be endorsed by GPs.

At the time of the interviews, websites trusted by doctors in Australia, such as the TGA website, had not included up to date information on e-cigarettes. This information is now available online and includes a short video describing the different pathways GPs can take to register to prescribe for e-cigarettes as well as information on the three pathways, product standards, prescription of e-cigarettes and temporary items added to the Medical Benefits Schedule associated with nicotine and smoking cessation [4]. Furthermore, guidance for the use of e-cigarettes for smoking cessation can be found [4, 40].

The RACGP have information available to GPs on the safety and efficacy of e-cigarettes, the three pathways, prescribing and writing prescriptions, dosing and device considerations, flavours, follow up consultations and monitoring use of e-cigarette products [13]. Information is also provided for specific groups including patients with chronic illnesses, pregnant or breastfeeding patients, adolescents, patients with mental illness and patients that are Aboriginal and Torres Strait Islander peoples [13].

The understanding of the current policy around prescription of e-cigarettes from the participants we interviewed was poor. This could be due to our participants not having had sufficient time to learn about and understand the changed policy guidance that occurred in 2021 a few months prior to our interviews. Those who were aware of the policy change voiced concerns about being gatekeepers to e-cigarettes and had concerns about their role in prescribing e-cigarettes. Some were frustrated with this decision and questioned why doctors were given this role and not other health professionals. Clearly there is a level of discomfort that needs to be addressed, and in particular clarity on indemnity and risks given the e-cigarette products are not currently approved by Australia’s therapeutic goods regulator, the TGA. Some of our participants were clearly confused by the information on the TGA website at the time, as no e-cigarette is TGA approved however, the RACGP mention the use of e-cigarettes as a second line treatment combined with behavioural support. Australian GPs need clear smoking cessation and prescribing guidelines that are easily accessible to them in a busy clinical environment. Furthermore, educational materials to provide to patients in their consultations (i.e. infographics, one-page summaries on pro’s and con’s) that explains the types of e-cigarette devices and nicotine liquids that can be used as part of a smoking cessation plan were desired.

### Limitations

The findings from this research cannot be generalised to all GPs as it is a small, heterogenous sample. It does however provide insights and clues to areas of clinical need and areas for future research. Population prevalence of smoking in Australia is amongst the lowest in the world and consistent with this, some participants hadn’t consulted with patients suitable to use e-cigarettes for smoking cessation. Their perceptions may differ from other GPs who treat higher volumes of patients for tobacco and nicotine dependence/addiction such as those who are opioid dependent or with mental health conditions, indigenous and patients in rural clinics.

## Conclusion

This is the first qualitative study of Australian GPs attitudes, knowledge and prescribing intentions with respect to e-cigarettes for smoking cessation. The participants we interviewed had diverse views about e-cigarettes for smoking cessation and is probably reflective of broader experience and perspectives within Australian general practice at this time. Concerns about safety and efficacy and limited understanding of current policy guidelines were strong influences on prescribing intentions. Participants voiced concerns about the possibility of e-cigarettes being a gateway to smoking and other tobacco products especially, amongst young adults and the potential for dual use. Limited confidence to have discussions and answer patient questions about e-cigarettes arose from insufficient knowledge and concerns about the veracity of scientific evidence about e-cigarettes. Australian GPs are concerned about their role as gatekeepers to e-cigarettes and the potential risks to patients. Clearer guidance, which is easily accessible to GPs is required.

### Electronic supplementary material

Below is the link to the electronic supplementary material.

Supplementary Material 1

## Data Availability

The datasets used and/or analysed during the current study are available from the corresponding author on reasonable request.

## Abbreviations

COREQ: Consolidated Criteria for Reporting Qualitative Research
E-Cigarettes: Electronic Cigarettes
ENDS: Electronic Nicotine Delivery Systems
GPs: General Practitioners
NRTs: Nicotine Replacement Therapies
RACGP: Royal Australian College of General Practitioners
TGA: Therapeutics Good Australia
TPB: Theory of Planned Behaviour
