# Supplementary material for: ‘Why do we have to be the gatekeepers?’ Australian general practitioners’ knowledge, attitudes and prescribing intentions on e-cigarettes as a smoking cessation aid
Source: BMC Prim Care. 2024 Feb 7;25:53. doi: 10.1186/s12875-024-02292-w (PMC10848430; doi:10.1186/s12875-024-02292-w)
Supplement: Supplementary file 1 — Supplementary Material 1 [file 12875_2024_2292_MOESM1_ESM.docx]

**Supplementary File**

S1. Interview guide

**Introduction and general information**

Theme 1. Firstly, can you tell me a little about your clinical practice and the patients you see?

Probe: How long have you worked at this clinic?

Probe: Do you have any fields of special interest?

**Current practice on smoking cessation**

Theme 2. Of your patient group, do you have many who are smokers? Tell me a bit about them.

Probe: How do you identify them?

Probe: Is smoking status routinely recorded in practice software.

Is there any one thing about a patient or their smoking that will lead you to bring up the topic of smoking cessation?

Probe: Are GPs best to provide smoking cessation counselling, or do you refer to other organisations like QUIT?

Probe: What types of smoking cessation methods do you recommend to help your patients quit smoking?

Probe: How confident do you feel providing smoking cessation advice?

Probe: What would be needed for you to feel more confident?

**Patient discussions**

Theme 3. Of your patient group, do you have many who use e-cigarettes or are vaping? Tell me a bit about them.

Probe: What types of discussions have you had with patients about e-cigarettes?

Probe: Can you give examples of what you would say if you were asked about e-cigarettes?

Probe: Are GPs best to provide counselling about e-cigarettes or do you refer to other organisations like QUIT?

Can you give me an example of when a patient asked you about vaping, or perhaps, when you spoke with a patient about their vaping?

**Beliefs about e-cigarettes**

Theme 4. There have been calls from some groups, including professional groups, that e-cigarettes be considered for use to aid smoking cessation.

What are your overall thoughts about e-cigarettes as a smoking cessation aid?

Probe: How do you think e-cigarettes compare to other available smoking cessation aids?

Probe: What reasons make you think this or why do you think this?

Probe: What do you think about the relative harms, risks and safety of e-cigarettes compared to smoking regular cigarettes?

Probe: What are your reasons for thinking this or why do you think this?

Probe: What types of concerns do you have about the use of e-cigarettes as a cessation aid?

Probe: What are your opinions about e-cigarettes potentially triggering dual use?

Probe: What are your thoughts about e-cigarettes causing a gateway effect to smoking and other tobacco products?

**Attitudes towards the use of e-cigarettes as a smoking cessation aid**

Theme 5. What types of patients would you recommend e-cigarettes to?

Probe: What is your reason for recommending them to the types of patients you suggested? (i.e. anything specific about the patient? (e.g. COPD, CVD patients)

If you would NOT recommend them, why would you not recommend them, what is your reasoning for this?

Probe: What would change your opinion on e-cigarettes to recommend them? (i.e. more research, information?

**Knowledge about e-cigarettes**

Theme 6. Do you feel you have enough knowledge on e-cigarettes to confidently answer patients’ questions?

If Yes, where do you receive e-cigarette information from and what types of evidence do you rely on?

Probe: What other information and guidance would you like to see on e-cigarettes to help you in your current practice?

If No, what information and guidance would you like to see on e-cigarettes to help you in your current practice?

**Prescribing and policy on e-cigarettes**

Theme 7. What is your understanding of current policy and guidance on e-cigarettes in Australia?

Probe: How much does current policy and guidance influence the advice you give to patients about e-cigarettes?

Probe: What other groups have influenced your advice and decision to give patients about e-cigarettes? i.e. colleagues, family members, friends, other organisations.

Probe: How do you feel about the current prescribing laws in Australia around e-cigarettes?

Probe: Do you feel you know enough to comfortably prescribe e-cigarettes to your patients or do you feel as if you require further information and training in this area?

Probe: Do you think it is best for GPs or for others such as respiratory nurses to counsel and/or prescribe e-cigarettes to patients for smoking cessation?

**Confidence and comfort about e-cigarette advice**

Theme 8. How supported/comfortable/confident do you feel in giving advice about e-cigarettes to your patients?

Probe: What other support services, programs or information would you like to see from

- the government regarding e-cigarettes as smoking cessation aids
- The RACGP
- Other health promotion groups, like Cancer Council, or QUIT organisations

Finally, do you have any other issues or concerns around e-cigarettes as smoking cessation aids that you wish to raise?
